# Toll-like receptor agonists partially restore the production of pro-inflammatory cytokines and type I interferon in Sézary syndrome

**DOI:** 10.18632/oncotarget.12816

**Published:** 2016-10-21

**Authors:** Kelly C. G. Manfrere, Marina P. Torrealba, Denis R. Miyashiro, Luanda M. S. Oliveira, Gabriel C. de Carvalho, Josenilson F. Lima, Anna Claudia C. C. Branco, Nátalli Z. Pereira, Juliana Pereira, José A. Sanches, Maria N. Sato

**Affiliations:** ^1^ Department of Dermatology, Laboratory of Medical Investigation, LIM 56, Tropical Medicine Institute of São Paulo, University of São Paulo Medical School, Brazil; ^2^ Department of Dermatology, Cutaneous Lymphoma Clinic, Hospital das Clínicas, University of São Paulo, Medical School, Brazil; ^3^ Department of Hematology, University of São Paulo Medical School, Brazil

**Keywords:** Sézary syndrome, innate immunity, Toll-like receptor agonists, cytokines, type I interferon, Immunology and Microbiology Section, Immune response, Immunity

## Abstract

Sézary syndrome (SS) carries a poor prognosis, and infections represent the most frequent cause of death in SS patients. Toll-like receptors (TLRs) are a family of innate immune receptors that induce protective immune responses against infections. We sought to evaluate the ability of TLR agonists to induce inflammatory cytokine, Th2 cytokine, and type I interferon (IFN-I) production by peripheral blood mononuclear cells (PBMC) of untreated SS patients. We detected impaired IL-6, IL-10 and IL-13 secretion by PBMC induced by the agonists for TLR5, TLR3, TLR7 and TLR9 in SS patients, while it was partially recovered by TLR2/TLR4 and TLR7/8 agonists TNF secretion was restored following stimulation with TLR2/TLR4 agonists. IFN-γ was scarcely produced upon TLR activation in SS cells, albeit TLR 7/8 (CL097) enhanced their secretion at lower levels than the control group. TLR9 agonist efficiently induced IFN-I in SS patients, although this positive regulation was not observed for other cytokines, in direct contrast to the broad activity of CL097. Among the TLR agonists, TLR4 was able to induce pro-inflammatory, IL-10 and Th2 secretion, while TLR7-8 agonist induced the inflammatory cytokines, IFN-I and IFN-γ. These findings reveal a dysfunctional cytokine response upon both extracellular and intracellular TLR activation in SS patients, which was partially restored by TLRs agonists.

## INTRODUCTION

Cutaneous T-cell lymphomas (CTCL), is a non Hodgkin lymphoma that affects skin. The most common type of skin infiltration is mycosis fungoides (MF), with the clinical picture of patches and plaques that may evolve into tumors and erythroderma. Sézary syndrome (SS), is an aggressive CTCL with dissemination of malignant CD4+ T cells with a memory phenotype that can be found in skin, blood, and lymph nodes [1].

During disease progression, a variety of immunologic abnormalities are observed regarding cell-mediated immunity in SS patients [2–3], with decreased production of interferon (IFN)-γ (Th1 cytokine), and down-regulated expression of Th1-specific genes, such as TBX21 (T-bet), NKG7, and SCYA5 (RANTES) [2, 4]. Defects in the production of interleukin 12 (IL-12) [3] and increased Th2 cytokines secretion (IL-4, -5, -10, and -13) by the Sézary cell, which can antagonize with Th1 function, have also been reported [3–5]. Moreover, SS cells exhibit constitutive STAT3 phosphorylation [6–7]. The Jak3/STAT pathway promotes the expression of IL-5, IL-17A/F and IL-10, regulates the production of angiogenetic factors, and confers resistance to treatment with histone deacetylase inhibitors in malignant T cells [8–10].

Considering the altered profile of cytokines secretion in SS, the activation of innate immunity may represent a strategy to potentiate innate immunity and consequently adaptive responses. The enhancement of immunity in SS should be crucial to ameliorate protection against bacterial/viral infections considering that sepsis is common cause of death among SS patients. Moreover, the activation antigen presenting cells by TLRs may lead to an improvement of the adaptive response that is crucial to control tumor burden. The TLRs recognize the pathogen-associated molecular patterns (PAMPs) and have been targeted to treat a variety of skin cancers [11]. Signalling by TLRs involves adaptor proteins known as myeloid differentiation primary-response gene 88 (MyD88), MyD88-adaptor-like (TIRAP/Mal), TIR-domain-containing adaptor protein inducing IFN-β (TRIF) and TRIF-related adaptor molecule (TRAM) [12]. Between the TLR2 recognize lipopeptides, TLR4 recognizes lipopolysaccharide (LPS), and TLR5 flagellin. The TLR3, are involved in the recognition of double-stranded RNA, TLR7 and TLR8 recognize single-stranded RNA viruses, and TLR9 is crucial to recognized unmethylated CpG DNA. Human TLR7 and TLR8 recognize guanosine- or uridine-rich ssRNA as well as imidazoquinoline compounds [13–15]. After recognition of PAMPs, TLRs initiate downstream signaling events that culminate in cytokines secretion and initiation of the adaptive immune response.

The modulatory effect of TLR activation in the cytokines response in SS has been showed by the activation of peripheral blood mononuclear cells (PBMC) using the TLR7 agonist (3M-001), which induces increased levels of IFN-α production, and the TLR8 agonist (3M-002), inducing IL-12 and IFN-γ [16]. The combination of IFN-γ or IL-15 with the TLR7/8 agonist 3M-007 was shown to significantly increased Natural Killer (NK) cytotoxic response to CTCL cell lines. Indeed, the *in vitro* potency of TLR agonists has been translated into clinical benefit. Safe and effective results were observed with subcutaneous CpG (TLR9) [18–19], and topical resiquimod (TLR7/8) in the treatment of CTCL patients [20].

Here, we sought to determine the ability of TLR agonist family members to induce pro-inflammatory and Th2-related cytokine production by PBMC from SS patients. We found that the impaired pro-inflammatory cytokines, Th2/Th1 cytokines and IFN-I production in SS patient cells, were partially restored upon TLR2/TLR4 and TLR7/8 agonist agonists stimulation.

## RESULTS

### TLR agonists induce cytokine production in SS patient cells

The laboratory characteristics of SS patients are shown in Table S1. All patients were erytrodermic with intense pruritus, and 10/14 patients showed lymphocytosis. Although two patients (patients 5,16) were negative for T cell clones in two compartments (blood, skin or lymph nodes) evaluated, the hospital likely did not employ probes related to the clone present by these patients, whereas the clinical features together with laboratory data were essential to conclude the SS diagnosis.

**Figure 1:**
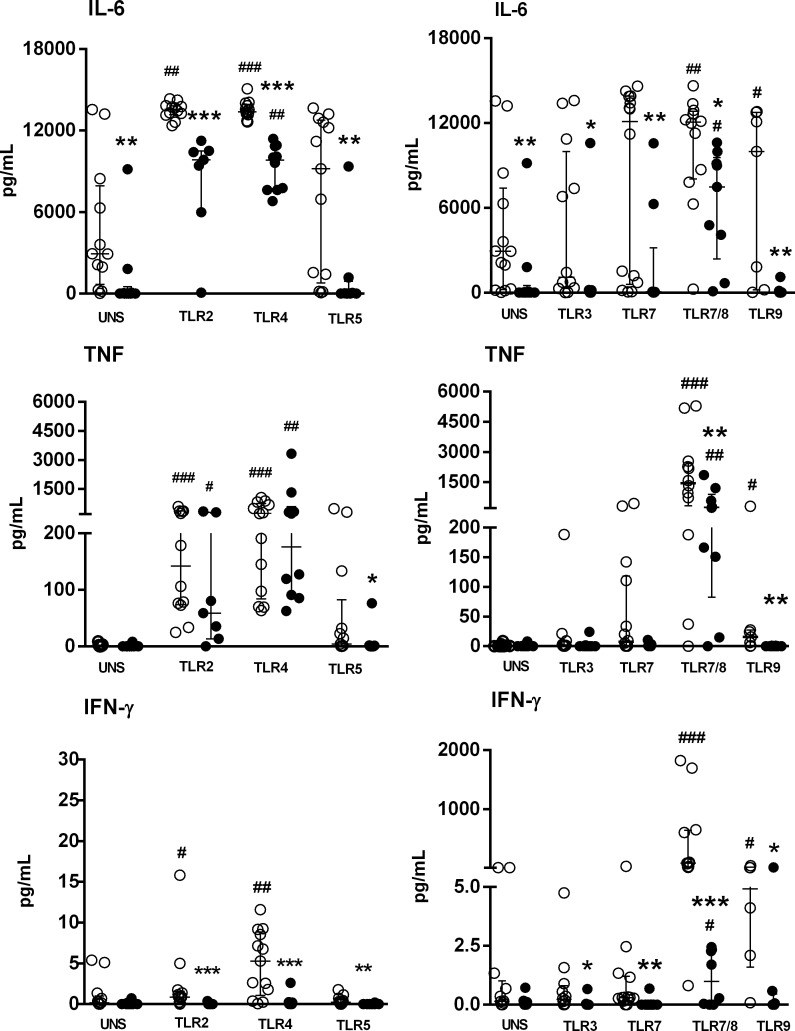
Impaired IL-6, TNF and IFN-γ production by PBMC of SS patients induced by TLR activation PBMC from SS patients (*n* = 10, closed circle) and healthy controls (HC, *n* = 13, open circle) were cultured in medium (unstimulated, UNS) or with TLR agonists (TLR2-TLR9) for 48 h. Supernatants were assessed for IL-6, TNF and IFN-γ secretion with a cytometric bead array. The results are shown as medians and interquartile ranges (IQRs).*p ≤ 0.05, **p ≤ 0.01, ***p ≤ 0.001 compared with the HC group; #*p* ≤ 0.05, ##*p* ≤ 0.01, ###*p*≤ 0.001 compared with the unstimulated culture.

At baseline levels (unstimulated PBMC), a decreased IL-6 secretion was detected in SS patients than HC subjects (Figure 1). Activation via TLR4 agonists induced high levels of IL-6 in the SS group, although these levels were lower than those noted in HC subjects (Figure 1). TNF secretion was restored by both TLR2 and TLR4 stimulation in the SS group, at the same level as HC subjects. The TLR5 agonist was not able to induce IL-6 and TNF secretion in both the HC and SS groups. For intracellular TLRs, the unique TLR agonist CL097 (TLR7/8) was able to induce IL-6 and TNF secretion in the SS and HC groups. The agonists of TLR5, TLR3, TLR7 and TLR9 did not induce IL-6 and TNF in the SS group.

An important impairment of IFN-γ secretion was noted in PBMC from SS patients in response to TLR ligands, and TLR7/8 agonist was able to induce IFN-γ, although it was lower than the control group. In comparison, for HC, IFN-γ in PBMC was triggered by TLR2, TLR4, TLR7/8 and TLR9 agonists (Figure 1).

**Figure 2:**
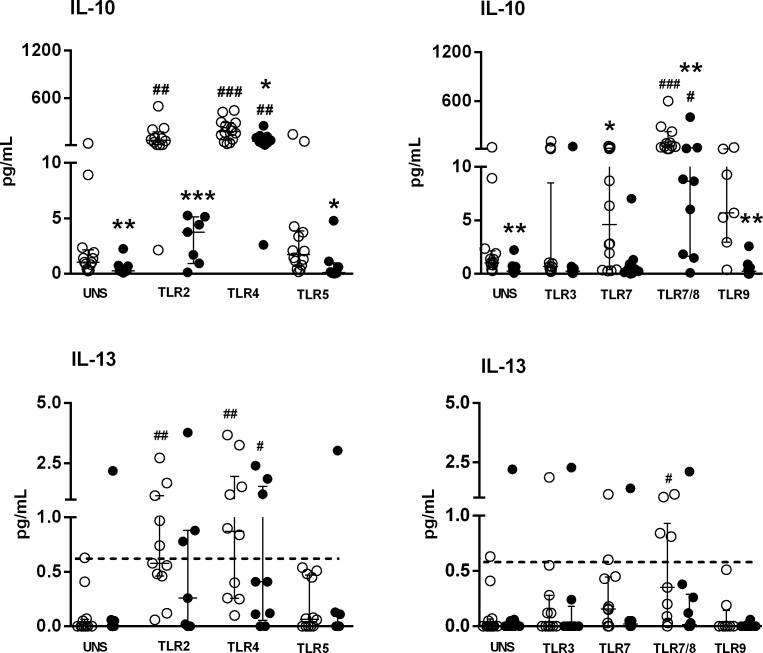
Altered IL-10 and IL-13 secretion by PBMC of SS patients induced by TLR agonists PBMC from SS patients (*n* = 10, closed circle) and healthy controls (HC, *n* = 13, open circle, open circle) were cultured with medium (unstimulated, UNS) or stimulated for 48 h with TLR agonists (TLR2-TLR9). Supernatants were assessed for IL-10 and IL-13 secretion with a cytometric bead array. Horizontal line shows the detection limit for IL-13. The results are shown as medians and IQRs.*p ≤ 0.05, **p ≤ 0.01, ***p ≤ 0.001 compared with the HC group; #*p* ≤ 0.05, ##*p* ≤ 0.01, ###*p* ≤ 0.001 compared with the unstimulated culture.

Moreover, the baseline IL-10 levels were decreased in SS patients. However, these levels were up-regulated upon activation of TLR4 and TLR7/TLR8 in both groups, albeit at lower levels in the SS group (Figure 2). Curiously and in contrast to IL-6 secretion, IL-10 secretion was not induced after TLR2 stimulation in SS patient cells, which was enhanced with a TLR2 agonist.

An enhanced sensitivity flex kit, which can detect fentogram/mL levels by flow cytometry, was used to asses Th2 cytokines (IL-4, IL-5). However, IL-4 and IL-5 were undetectable in the supernatants of PBMC from both HC and SS samples stimulated with TLRs agonists. Figure 2 shows the low-level secretion of IL-13 induced by TLR2, TLR4 and TLR7/8 agonists in the HC group, whereas only activation by TLR4 induced IL-13 in SS samples. These results show that TLR activation induced low levels of Th2 cytokines from PBMC in both analyzed groups.

We next sorted the CD4+CD158k+ T cells (malignant T cells) from SS patients to verify their ability to produce Th2 cytokines upon TLR activation. The sorting procedure was performed with only 3 SS patients (patients 2, 5 and 15) because most patients did not survive. Moreover, only patient 2 showed the presence of CD4+CD158k+ cells, which did not secrete Th2 cytokines upon TLR2/TLR4 or TLR7/8 stimulation.

IL-17A was also undetectable in the supernatants of PBMC activated by TLR agonists in both analyzed groups.

These results revealed an adjuvant effect of TLR2 and TLR4 agonists on IL-6, TNF, IL-10 and IL-13 secretion, while TLR7/8 agonists increased IL-6, TNF, IL-10 and IFN-γ production by PBMCs of SS patients.

### Intracellular TLR agonists induce IFN-α production in SS patient cells

**Figure 3:**
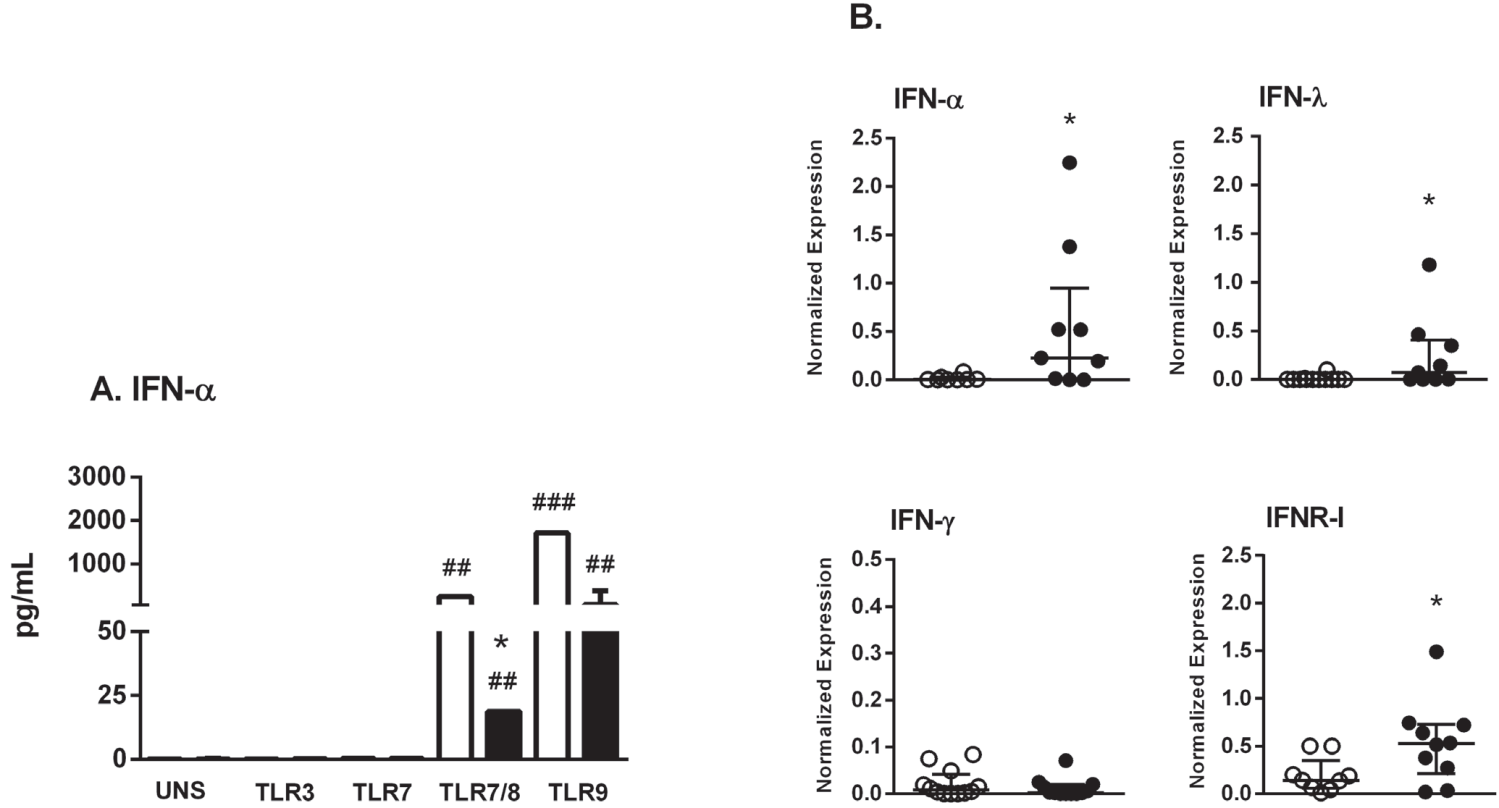
Agonists of TLR7/8 and TLR9 are able to induce IFN-I secretion in SS patient cells **A.** PBMC from SS patients (*n* = 11, closed bar) and healthy controls (HC, *n* = 13, open bar) were cultured with medium (unstimulated, UNS) or stimulated for 48 h with agonists of TLR3, TLR7, TLR7/8 or TLR9. Supernatants were assessed for IFN-α secretion with a cytometric bead array. **B.** PBMC from SS patients (closed circle, *n* = 9) and HC (open circle, *n* = 12) were assessed for IFN-α, IFNR-I, IFN-λ and IFN-γ mRNA expression by qPCR. The results are presented as medians and IQRs.*p ≤ 0.05 compared with the HC group; #*p* ≤ 0.05, ##*p* ≤ 0.01 compared with the unstimulated culture.

We detected IFN-α secretion by PBMC stimulated with TLR9 agonists as well as the TLR7/8 CL097 agonist, but not with TLR3 and TLR7 agonists (Figure 3a). Significant IFN-α secretion was induced following stimulation of PBMC from HC with the TLR9/CpGA agonist, and this level was 29-fold higher compared to that observed in SS patients. In addition, secretion in response to the TLR7/8 agonist was increased 13-fold in HC compared with SS subjects (Figure 3a). Next, we evaluated the profile of TLR mRNA expression in unstimulated PBMC.

Figure 1S shows the similar profile of TLR mRNA expression (TLR2,-3,-4,-7,-8 and -9) in PBMC from HC and SS subjects. Figure 3b presents IFN-I (IFN-α, receptor IFNR-I), II (IFN-γ) and III (IFN-λ) mRNA expression levels. As expected, no alterations in IFN-γ were detected between groups, whereas types I and III IFN were increased in SS subjects. At the time of the experimental analyses, no patient had previously received IFN-α treatment. Moreover, IFN-α was undetectable in the serum of SS patients and HC. The IL-6, TNF-α and IL-10 mRNA levels of unstimulated PBMC were similar in both groups (data not shown).

**Figure 4:**
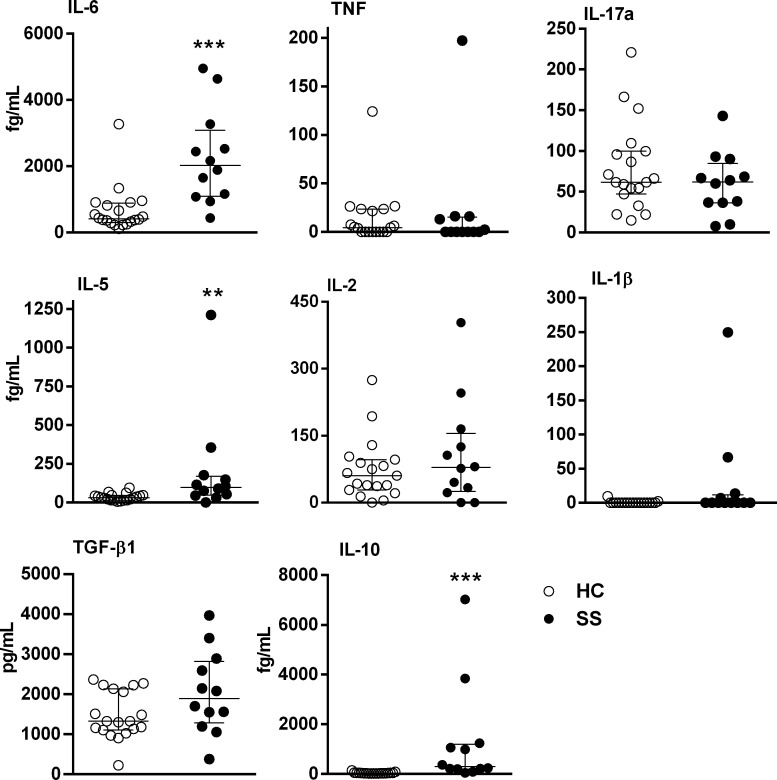
Cytokine serum levels in SS patients Sera from SS patients (*n* = 12, closed circle) and healthy controls (HC, *n* = 19, open circle) were assessed for cytokines using high-sensitivity flex sets for TNF, IL-6, IL-5, IL-10, IL-2 and IL-17A or a flex set for TGF-β1 by flow cytometry. The results are presented as medians and IQRs. **p ≤ 0.01, ***p ≤ 0.001 compared with the HC group.

Next, we assessed the IL-6, TNF, IL-17A, IL-5, IL-2, IL1β, TGF-β1 and IL-10 serum levels. It was detected high levels of IL-5, IL-6, and IL-10 in SS serum compared with HC serum (Figure 4).

## DISCUSSION

The study evaluated the profile of cytokine production by PBMC induced by TLR agonists in SS patients. Studies focusing on pro-inflammatory, Th2, as well as IFN-I production, in SS patients after stimulation with extracellular and intracellular TLR agonists have not been reported previously. Our findings reveal a dysfunctional cytokine response upon TLR activation in SS patients; indeed, the TLR2/TLR4 agonist was able to partially restore IL-6, TNF, IL-10 and IL-13 production, while TLR7/8 showed an adjuvant effect on IFN I and II, IL-10 and pro-inflammatory cytokine secretion.

Overall, we observed a different profile of cytokine secretion between extracellular and intracellular TLRs agonists, whereby they are more affected following intracellular TLR activation. To determine whether these differences may have been due to different levels of TLRs expression, we observed similar expression of TLRs mRNA levels in PBMC between HC and SS patients, despite the fact that up to 90% of SS lymphocytes represent malignant Sézary cells. This finding showed that the impairment of TLRs activation was not due to altered TLRs expression and may be related to a dysfunctional signaling pathway or epigenetics mechanism, such as hypermethylation of cytokine gene promoters. These mechanisms needs to be further evaluated in SS patients.

We observed that IL-6 and TNF secretion was partially restored by activation with TLR2/4 and TLR7/8 agonists, while the agonists of TLR5, TLR3, TLR7 and TLR9 were unable to induce these cytokines. Although TNF secretion was similar to the control group, IL-6 and IFN-γ production remained low and could possibly explain the susceptibility of SS patients to bacterial infection. In fact, in SS patients, sepsis with *Staphylococcus aureus* and *Pseudomonas aeruginosa* can be caused by chronic skin infections and subsequent systemic infections [21].

Differential IL-10 responses were observed by TLRs ligands in SS patients, which were weakly induced by TLR2 and restored by TLR4 agonists, albeit at lower levels than healthy controls. It has been described that TLR2 activation induce a degradation of IL-10 mRNA while TLR4 activation preserves IL-10 mRNA due to the activation of TRIF and p38 signaling, in mouse system [22].

The serum levels of IL-6 in SS patients were increased 4.85-fold compared with those in HC. In SS patients, the levels of neopterin, ß2-MG and sIL-2R are significantly elevated, and IL-6 correlates with tumor burden [23]. CTCL tumor cell lines, but not non-malignant cell lines, produce IL-6 spontaneously [10]. Constitutive STAT5 activation is detected in early stage of CTCL and STAT3 in late stage of disease [24]. Moreover, STAT5 and STAT3 activation could be triggered by constitutively active JAK1 and JAK3 [24]. ur cohort of However, there was no association between cytokine serum levels and the prominent laboratory findings in SS. Moreover, although increased IL-6 serum levels were detected in SS, the baseline secretion levels of PBMC were decreased. This is probably a consequence of different sources of secreting cells, since several non-immune cells can contribute to this secretion in the serum. A similar effect was detected with IL-10 serum levels and secreted by PBMC in SS.

The etiology of CTCL remains uncertain, and samples have been analyzed for retroviral or other viral causes. However, studies have failed to show associations between viruses and CTCL [25–27]. Notably, we observed an impaired response to agonist treatment via TLR3, TLR7, TLR8 and TLR9 in our SS patients, although activation could be strategically overcome with some TLR agonists. CL097, a derivative of the imidazoquinoline (IMQ) compound R848, recovered IL-6, TNF, and IL-10 as well as IFN-I production by PBMC from SS patients. Given that TLR7 activation does not induce cytokine production, this finding suggests that TLR8 activation overcame the impaired cytokine secretion. TLR8 agonist may overcome the inhibitory function of adenosine/cAMP avoiding fully dendritic cells activation [28]. Thus, the adjuvant effect of CL097 must be further evaluated, although our previous reports also demonstrated improvement in cytokine production induced by CL097 in cutaneous diseases, such as lichen planus [29], and during HIV infection [30].

Although IFN-α secretion levels in SS were restored with the TLR9 agonist (CpGA), this positive regulation was not observed for other cytokines, in direct contrast to the broad activity of CL097. These different effects could be due to the distinct signaling pathways triggered by these agonists. IFN-I production stimulated byCpG-A is mediated by PI3K and mTOR [31], whereas TLR7 and TLR8 activate IRF5 and IRF7 [32]. Curiously, upon evaluating type I and type III IFN expression in PBMC, some patients exhibited a constitutive increase in these IFNs (patients 1, 4 and 9) that was not due to seropositivity for viral infections.

Impaired cell-mediated immunity and decreased production of IFN-γ may be due to IL-12 deficiency [33] or a Th2 cytokine profile inhibiting the Th1 response in CTCL patients [9]. Again, the adjuvant effect of CL097 was observed for the IFN-γ response of 50% of SS patients, and very low levels were noted compared with the HC subjects.

The continuous production of IL-4, IL-5, and IL-10 by Sézary cells could represents evasion mechanism to tumoral immune response [18]. Thus, the production of Th2 cytokines might promote disease progression, given that Th2 cytokines may inhibit the antitumor effect. bias of leukemic Sézary cells toward the Th2 phenotype [34], we did not observe enhanced Th2 secretion by PBMC from SS patients (10 patients) or by malignant Sézary cells derived from one patient. Following TLR stimulation, IL-4 and IL-5 secretion were undetectable in either patients or controls, which indicates that the response to PAMPs is mainly related to Th1 rather than Th2 cytokines. The TLRs activation is crucial to induce stimulation of IL-12p70 and IFN-α secretion from DC and driving TLR-mediated Th1 responses [35]. In fact, very low IL-13 secretion levels by PBMC were detectable upon TLR2/TLR4 and TLR7/8 by healthy control; in contrast, these were almost absent in SS. This finding shows that, despite the known Th2 phenotype of malignant T cells in SS patients upon TLR activation, the mononuclear cells are not able to produce Th2 cytokines.

Together, our data reveal an impaired cytokine response to TLR signaling in SS patients. The decreased TLR response in cytokine production, including pro-inflammatory cytokines and IFN-I, was partially reversed with an agonist of TLR2, TLR4 and TLR7/8, suggesting its adjuvant role in SS.

## MATERIAL AND METHODS

### Casuistic

Our study included patients with Sézary syndrome (SS; *n* = 14, 7 males, 7 females), with a median age of 61 years (range 48-75 years), from the Clinic of Cutaneous Lymphomas of the Department of Dermatology, Hospital das Clínicas, University of São Paulo Medical School, Brazil (HC/FMUSP). The SS diagnosis was established based on recognized international clinical, histological, and biological criteria proposed by the World Health Organization (WHO) and European Organization for Research and Treatment of Cancer (EORTC) [36]. Patients were not previously treated with immunomodulating agents or chemotherapy. Healthy controls (HC; *n* = 19, 8 male, 11 female), with a median age of 58 years (range 33-85 years), were selected from the Laboratory of Medical Investigation, LIM56.. Blood collection was performed before the initiation of treatment. Patients with dermatologic diseases, those using drugs, and those with a history of autoimmune diseases were not included in this evaluation. xclusion criteria consisted of treatment with immunosuppressant or immune-modifying drugs, pregnancy, and less than 18 years of age. This study was approved by the São Paulo University Institutional Use Committee (CAAE, 07965312.0.0000.0068), and informed consent was obtained from all subjects. All experimental protocols within this study were performed in accordance with the Ethics Committee of this institution.

### Cell culture

PBMC were isolated from heparinized venous blood by Ficoll-Hypaque gradient centrifugation (GE Healthcare, Uppsala, Sweden) and diluted in RPMI medium supplemented with 10% AB human serum (Sigma, St Louis, MO,U.S.A.). PBMC cultures (2.0 × 10^5^cells/well) were incubated in 96-well microplates (Costar, Cambridge, MA, U.S.A.) in medium in the presence of ligands for TLR2 (pam3csk4, 0.5 gmL^−1^); TLR3 [poly(I:C)-RIG, 20 ng mL ^−1^]; TLR4 (LPS, 1.25 μg mL ^−1^); TLR5 (flagellin, 0.5 ng mL ^−1^); TLR7 (imiquimod, 1.25 gmL^−1^); TLR7/TLR8 (CL097, 2.5 μg mL^−1^) and TLR9 (CpGA 2206,4 mol L^−1^) for 48 h at 37°C and 5% CO_2_. All ligands were obtained from InvivoGen (San Diego, CA, U.S.A.). Cell-free supernatants were harvested and stored at −80°C until cytokine measurements were obtained with a cytometric bead array.

### Cytokine measurements

Cytokine levels in the serum were assessed using enhanced sensitivity flex kits for IL-5 (limit of detection 67.8 fg/mL), IL-6 (68.4 fg/mL), IL-10 (13.7 fg/mL),IL-17a (16.1 fg/mL), TNF (67.3 fg/mL), IL-2 (88.9 fg/mL),IL-1β (48.4 fg/mL) and IL-2/TGF-b1 (14.9 pg/mL) from BD Biosciences (CA, USA). Cell culture supernatants were analyzed using cytokine kits to measure IL-6 (2.4 pg/mL), IL-10 (4.5 pg/mL), IL-13 (0.6 pg/mL), TNF (3.8 pg/mL), IFN-γ (3.7 pg/mL) and IFN-α (1.5 pg/mL) or enhanced sensitivity flex kits for IL-4 (144.4 fg/mL), IL-5 (67.8 fg/mL) and IL-17A (26.1 fg/mL). Samples were assessed using flow cytometry (LSR Fortessa, BD, San Jose, CA, U.S.A.).

### Real-time PCR

Total RNA was extracted from PBMC using an RNeasy Plus Mini Kit (Qiagen, Valencia, CA, USA), and reverse transcription was performed with a with a iSCRIPT reverse transcriptase kit (Biorad, California, EUA). The primers used in the real-time PCR assay are detailed in Table S2.

GAPDH mRNA levels were used to normalize the mRNA content from PBMC. PCR was performed on an Applied Biosystems 7300 system using specific primers and SYBR Green (Applied Biosystems, Carlsbad, CA, USA) fluorescence detection reagents. The cycling protocol consisted of 10 min at 95°C, followed by 40 cycles of 15 s at 95°C and 60 s at 60°C. The amplification results were visualized and analyzed using Sequence Detection System (SDS) software (Applied Biosystems). Normalized expression was calculated as previously described by Livak [37].

### Statistical analysis

The Mann-Whitney U-test was used to compare variables between the groups. Wilcoxon matched pairs test was used to compare baseline level with stimulated sample. *p* ≤ 0.05 was considered significant.

## SUPPLEMENTARY MATERIALS FIGURES AND TABLES


