# Supplementary material for: Toll-like receptor agonists partially restore the production of pro-inflammatory cytokines and type I interferon in Sézary syndrome
Source: Oncotarget. 2016 Oct 21;7(46):74592–601. doi: 10.18632/oncotarget.12816 (PMC5342689; doi:10.18632/oncotarget.12816)
Supplement: Supplementary file 1 [file oncotarget-07-74592-s001.pdf]

Toll-like receptor agonists partially restore the production of pro-inflammatory cytokines and type I interferon in Sézary syndrome

Supplementary Material

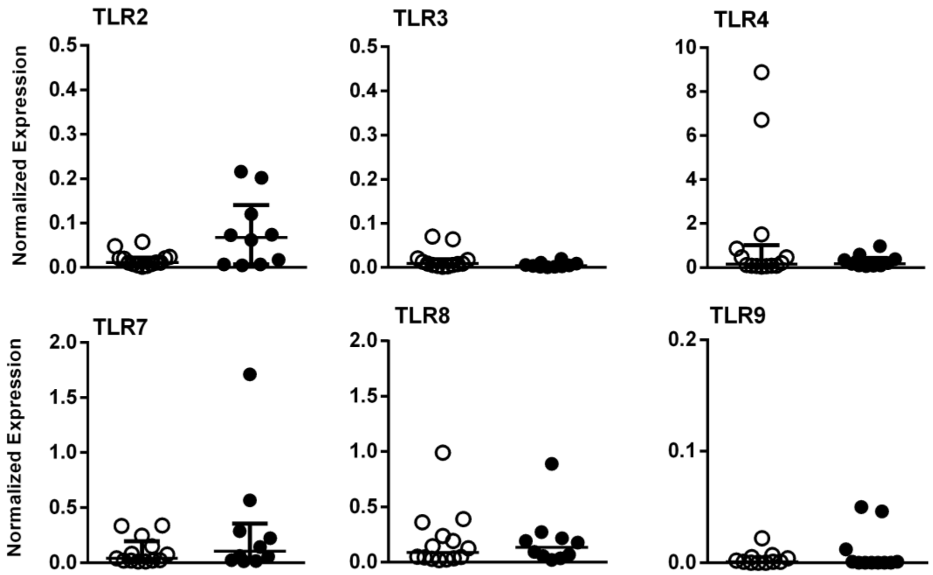

Figure 1 S. TLR mRNA expression in PBMC from SS patients and HC. The expression levels of TLR2, TLR3, TLR4, TLR7, TLR8 and TLR9 were analyzed in non-stimulated PBMC from SS patients (n=12, closed circle) and HC (n=15, open circle) by real-time PCR. The results are presented as medians and IQRs.

Table S1. Sézary Syndrome patients laboratorial characteristics.

Pat N°.	Gender	Age	lymph. /mm³	eosin. /mm³	Sézary/ mm³	CD4 /mm³	CD8 /mm³	CD4 /CD8	%CD4+ CD7-	%CD4+ CD26-	Tcell clone b/s/l	LDH /ref.
**1**	F	75	10200	650	+	9690	204	47.5	nd	75	+/+/+	1.2
**2**	F	55	21370	260	5128	20515	427	48	97	98	+/+/nd	1.2
**3**	M	56	980	300	-	823	29	28	3	36	+/nd/nd	1.6
**4**	M	70	2200	1100	264	2068	66	31	nd	90	+/-/nd	1.6
**5**	F	62	2030	750	+	1705	182	9.3	nd	52	-/-/nd	1.4
**7**	M	57	3490	50	419	3176	174	18	82	74	+/+/+	1.7
**8**	M	68	6830	150	1639	6251	295	22	81	86	+/nd/+	1.0
**9**	F	69	3070	270	-	2394	888	6	23	81	+/+/nd	1.0
**10**	M	56	7640	340	3667	6647	153	43.5	nd	79	+/+/+	1.8
**11**	M	62	8670	0	+	8150	347	24	41	52	+/+/+	2.5
**13**	M	48	21430	10270	+	20572	428	48	2	22	+/+/+	3.4
**15**	F	60	131010	1410	+	104808	0	-	100	100	+/+/+	1.7
**16**	F	65	3700	160	+	3293	148	22.2	88	82	+/-/-	1.8
**17**	F	70	7370	0	1695	6706	147	45.5	62	89	+/+/nd	1.8

Reference values for: lymphocytes: 0.9 – 3.4/mm^3; eosinophils: 0.05-0.5/ mm^3; CD4 /mm^3: 548-1724; CD8 /mm^3 : 330-1460; T cell clone blood/skin/lymph node: lactate dehydrogenase (LDH) ; ratio of sample value: maximum reference value; M:male. F: female. nd: not done.

Table S2.Sequences of the primers used for qPCR.

Gene	**FW primer**	**REV primer**
IFN-α	5'AAATACAGCCCTTGTGCCTGG 3'	5'GGTGAGCTGGCATACGAATCA3'
IFNR-I	5'GGCTGGCCCTGTGATATTTCTGTG3'	5'ACCTGGCTCTCCTCCTCCCTTCCT3'
IFN-λ	5'CGCCTTGGAAGAGTCACTCA3'	5'GAAGCCTCAGGTCCCAATTC3'
IFN- γ	5'TTTCATGCCTGGTGCTTCCA3'	5'GCTAAGAAGACTCCCCTCCCTA3'
TNF- α	5'CCCAGGCAGTCAGATCATCTTC3'	5'GCTTGAGGGTTTGCTACAACAT3'
IL-10	5'CAGGGCACCCAGTCTGAG3'	5'CACATGCGCCTTGATGTCT3'
IL-6	5'CCTGAGAAAGGAGACATGTAA3'	5'GGCAAGTCTCCTCATTGAATCC3'
TLR2	5’TCAGCCTCTCCAAGGAAGAA3’	5’AATGTTCAAGACTGCCCAGG3’
TLR3	5’GTGCCAGAAACTTCCCATGT3’	5’TCCAGCTGAACCTGAGTTCC3’
TLR4	5’CAGAGTTTCCTGCAATGGATCA3’	5’GCTTATCTGAAGGTGTTGCACA3’
TLR7	5’AATGTCACAGCCGTCCCTAC3’	5’GCGCATCAAAAGCATTTACA3’
TLR8	5’TGTGATGGTGGTGCTTCAAT3’	5’ATGCCCCAGAGGCTATTTCT3’
TLR9	5’GGGAGGAAGCTGCTAAGCTC3’	5’CTGGGAAGGACCAAGACCAC3’
